# The Pavlik harness in the treatment of developmentally dislocated hips: results of Japanese multicenter studies in 1994 and 2008

**DOI:** 10.1007/s00776-013-0432-z

**Published:** 2013-06-29

**Authors:** Ikuo Wada, Eisuke Sakuma, Takanobu Otsuka, Kenjiro Wakabayashi, Kinya Ito, Osamu Horiuchi, Yoshimi Asagai, Makoto Kamegaya, Eiji Goto, Shinichi Satsuma, Daisuke Kobayashi, Susumu Saito, Mayuki Taketa, Kazuharu Takikawa, Yasuharu Nakashima, Tadashi Hattori, Shigeru Mitani, Akifusa Wada

**Affiliations:** 1 Department of Functional Anatomy and Department of Orthopedic Surgery, Nagoya City University Graduate School of Medical Sciences, 1 Kawasumi, Mizuho-cho, Mizuho-ku, Nagoya, 467-8601 Japan; 2 Shinano Handicapped Children’s Hospital, Shimosuwa, Nagano Japan; 3 Chiba Children’s Hospital, Chiba, Japan; 4 Toyooka Chuo Hospital, Hokkaido, Japan; 5 Hyogo Prefectural Kobe Children’s Hospital, Kobe, Japan; 6 Showa University Fujigaoka Hospital, Yokohama, Japan; 7 Saga Handicapped Children’s Hospital, Saga, Japan; 8 Shizuoka Children’s Hospital, Shizuoka, Japan; 9 Kyushu University Hospital, Fukuoka, Japan; 10 Aichi Children’s Health and Medical Center, Obu, Japan; 11 Kawasaki Medical School Hospital, Kurashiki, Japan; 12 Fukuoka Children’s Hospital, Fukuoka, Japan

## Abstract

**Background:**

It has already been more than 50 years since the Pavlik harness was introduced in Japan, and today the Pavlik harness is widely recognized as the standard initial treatment modality for developmental dysplasia of the hip. We performed a multicenter nationwide questionnaire study concerning the results of Pavlik harness treatment twice in 1994 and 2008.

**Methods:**

In 1994 and in 2008, we sent questionnaires to 12 institutes in Japan specializing mainly in pediatric orthopedics. We compare the results of these two studies and discuss differences in reduction rates, incidence of avascular necrosis in the femoral epiphysis and the percentage of joints with acceptable morphology (Severin grade I + II/total) at skeletal maturity. We statistically assessed these results to see whether there were changes in the treatment outcomes over this 14-year period.

**Results:**

Reduction of the dislocated hips was obtained by the Pavlik harness in 80.2 % (1990/2481 hips; 1994) and 81.9 % (1248/1523 hips; 2008). The incidences of avascular necrosis of the proximal femoral epiphysis in the dysplastic hips were 14.3 % (119/835 hips; 1994) and 11.5 % (76/663 hips; 2008). The type of avascular necrosis in hips from the 2008 study was determined according to the classification of Kalamchi and MacEwen: 24/69 hips (34.8 %) were classified as group I; 20/69 hips (29.0 %) as group II; 11/69 hips (15.9 %) as group Ill; 14/69 hips (20.3 %) as group IV. The percentages of hips with acceptable outcomes at skeletal maturity discerned from Severin X-ray changes (grade I + II/total) were 72.3 % (604/835 hips; 1994) and 77.7 % (488/628 hips; 2008).

**Conclusion:**

Reduction rates and the incidence of avascular necrosis in 2008 were statistically similar to the results in 1994. The rate of acceptable outcome (Severin grade I + II/total) in 2008 was statistically higher than that of 1994.

## Introduction

Arnold Pavlik began to develop a functional harness for the management of developmental dysplasia of the hip in 1944, and Pavlik and his co-workers [1] presented the theory and their methods for the first time at the conference of the Czechoslovakian Society for Orthopedic Surgery and Traumatology in Prague in 1946. Pavlik harness treatment was introduced to Japan before 1960. Today the Pavlik harness has already gained widespread use throughout the world including Japan for the treatment of developmental dysplasia of the hip. The Pavlik harness induces spontaneous abduction of hips and a decrease in adductor muscle tone, leading to the gentle and spontaneous reduction and stabilization of the dislocated hip joints. If this harness is properly applied, a high ratio of hip reduction can be expected with a low complication rate. Previous reports have documented the favorable success rates of Pavlik harness treatment for developmental dysplasia of the hip. In a well-known study in Japan, Suzuki [2] reported a success rate of 182/216 hips (84.2 %) with 9 showing some residual deformity of the femoral head.

When hip reduction fails under Pavlik harness treatment, the hips usually require other treatment methods, such as manipulation under anesthesia and immobilization in a cast. These treatments result in an increased rate of avascular necrosis of the proximal femoral epiphysis [3, 4].

One of the most important review articles addressing the treatment of developmental dysplasia of the hip using the Pavlik harness was the publication in 1988 of the results of a comprehensive multicenter study of the European pediatric orthopaedic society by Grill and co-workers. They assessed the results of treatment of 3611 hips in 2636 children [5]. The children in this report were treated during the period 1976–1986 and followed from 1 to 8.9 years. The average age at the time of the initial treatment was 4.1 months (range 2 days to 11 months). In Grill’s report, treatment with the Pavlik harness achieved normal hips in 95.35 % of patients with dysplastic hips and 80 % of patients with dislocated hips. Overall, 92 % of all dislocated hips were reduced in the Pavlik harness, while the total rate of avascular necrosis of the proximal femoral epiphysis was 2.38 %.

In 1994 and in 2008, we sent questionnaires to multiple pediatric orthopedic institutions nationwide. Our points of interest were the reduction rate and incidence of avascular necrosis of the femoral epiphysis, and in children followed to skeletal maturity at age 14 years, the X-ray outcomes. We believe these statistical data should be an important benchmark for the treatment of dysplastic hips using the Pavlik harness.

## Patients and methods

First in 1994 and then in 2008, we sent questionnaires to 12 institutes in Japan specializing mainly in pediatric orthopedics. We requested data concerning children treated with the Pavlik harness for developmental dysplasia of the hip. Reduction rates, incidence of avascular necrosis in the femoral epiphysis and long-term outcomes were tabulated. Only hips with complete dislocations were included. For those children who had reached skeletal maturity (age 14 years), Severin grades were requested. Patients who underwent surgical procedures such as osteotomies were excluded. At the 12 institutions, a total of 2481 hips were initially treated with the Pavlik harness for dislocation in 1994, and 1523 hips were treated in this way in 2008. Accurate assessment of avascular necrosis was available for 835 hips in 1994 and 663 hips in 2008. Among those joints, 835 were followed to skeletal maturity and Severin grouping in 1994, with 628 hips similarly followed up in 2008.

Diagnosis of avascular necrosis was established from X-ray findings. In addition, hips displaying avascular necrosis of the femoral heads in 2008 were classified according to the classification of Kalamchi and MacEwen [6]: group I hips had changes affecting the ossific nucleus, group Il hips had lateral physeal damage, group III hips had central physeal damage, and group IV hips had damage to the entire femoral head and physis. Hips that were followed up to 14 years of age were evaluated according to Severn’s criteria as modified by Kasser et al. [7].

For the totaled results of multiple institutes in Japan, we made statistical analyses using the chi-square test concerning success rates of reduction by the Pavlik harness treatment between the two results in 1994 and 2008. We also made the same statistical analysis on the incidence of avascular necrosis of the femoral head and the rate of acceptable Severin X-ray changes (group I and II). Statistical significance was defined as a *p* value <0.05.

### Ethics statement

This study was approved by the institutional review board of Nagoya City University Graduate School of Medical Sciences according to the ethical guidelines for epidemiology research (approval no. 772).

## Results

The average age of the patients at the initiation of treatment was more than 3 months after birth in all institutions (range 3.5–4.8 months).

The reduction rates for the dislocated hips were 80.2 % (1990/2481 hips; 1994) and 81.9 % (1248/1523 hips; 2008). These results showed no statistically significant difference; *p* = 0.05 (Table 1).

**Table 1:** Reduction rate

Years	Hips	Reduction rate	%	Chi-square test
1994	2481	1990/2481	80.2	χ^2^ = 1.834
2008	1523	1248/1523	81.9	NS *p* = 0.05

The incidence of avascular necrosis of the proximal femoral epiphysis was 14.3 % (119/835 hips; 1994) and 11.5 % (76/663 hips; 2008). These results were also not significantly different; *p* = 0.05 (Table 2).

**Table 2:** Avascular necrosis of the proximal femoral epiphysis

Years	Hips	AVN	%	Chi-square test
1994	835	119/835	14.3	χ^2^ = 2.538
2008	663	76/663	11.5	NS *p* = 0.05

Classification of the type of avascular necrosis according to Kalamchi and MacEwen [6] was possible for 69 hips in 2008: 24/69 hips (34.8 %) were classified as group I, 20/69 hips (29.0 %) as group II, 11/69 hips (15.9 %) as group III and 14/69 hips (20.3 %) as group IV (Table 3).

**Table 3:** The classification of Kalamchi and MacEwen

Year 2008	Group I	II	III	IV
Total: 69 hips	24 hips; 34.8 %	20 hips; 29.0 %	11 hips; 15.9 %	14 hips; 20.3 %

The percentages of hips with acceptable X-ray outcomes at skeletal maturity (Severin grade I + II/total) were 72.3 % (604/835 hips; 1994) and 77.7 % (488/628 hips; 2008). Statistically, outcomes were better in 2008 than in 1994; *p* < 0.05 (Table 4).

**Table 4:** Sufficient rate for Severin grade (I + II/total)

Years	Hips	Severin I + II/total	%	Chi-square test
1994	835	604/835	72.3	χ^2^ = 5.464
2008	628	488/628	77.7	2008 > 1994 *p* < 0.05

## Discussion

Arnold Pavlik published 5 articles about his harness, its principles and his results from 1950 to 1959. He first reported the effectiveness of his orthotic device constructed of leather straps in 1950 [8, 9]. His most famous article was reported in the Zeitschrift fur Ortopaedie in 1957 [3], and this article was translated by Peltier [4]. He reported the results of 1912 hips (640 dysplasias, 640 subluxations and 632 dislocations) in 1424 children functionally treated with the Pavlik harness. He reported that no spontaneously reduced hips developed avascular necrosis of the femoral head. However, hips not spontaneously reduced were treated with passive-mechanical manipulation, and avascular necrosis occurred in 18 hips.

His article in 1959 was entitled “To the question of originality of treatment of congenital dysplasias of the hip joint by active movements in the stirrups” [10, 11]. In this article, he suggested that the main condition for success is the early start of treatment, preferably no later than 8 to 9 postnatal weeks.

Reasons for the failure of reduction in the Pavlik harness have been addressed by many investigators. Some have proposed a close relationship between age at initiation of treatment and the success rate of spontaneous reduction of dislocated hips. All of them have suggested that the earlier the treatment is started, the better results can be achieved. Viere and co-workers [12] reported that a child aged more than 7 weeks at initial treatment ran a significantly increased risk of failure for the stable reduction of hips. Harding and co-workers showed that the age at initiation of treatment influences the outcome of a 3-week trial of Pavlik harness treatment for reduction. They reported that infants who started the treatment with the harness after the age of 3 weeks had a higher failure rate (80 %) compared with those seen earlier (36 %) [13]. Atalar and co-workers reported that a statistical difference was found between successful and unsuccessful Pavlik harness treatment for the reduction of hips in terms of the infants’ age at the start of treatment. Infants aged 7 weeks and under had a higher rate of success than those aged 8 weeks and over [14].

Several investigators in Europe have also pointed out a close relationship between age at initiation of treatment and incidence of avascular necrosis of the proximal femoral epiphysis. In Grill’s report, statistical analysis showed that if the treatment was started within the first 3 months of life, the rate of avascular necrosis was markedly suppressed. The rate of avascular necrosis of the proximal femoral epiphysis was 50 % lower in these children than that in those who started treatment between the third and sixth months of life [5]. Kruczynski found that if treatment was started after the sixth month of life, the appearance of avascular necrosis of the proximal femoral epiphysis in the affected hip was significantly high [15]. Pap and co-workers [16] grouped their patients according to the ages at the start of treatment, and they found a close relationship between age and incidence of avascular necrosis of the proximal femoral epiphysis on the dysplastic side.

However, some investigators have suggested that there was no significant relationship between the patients’ age at initiation of treatment and either the reduction rate of dislocated hips or the incidence of avascular necrosis. Lerman and co-workers [17] reported a statistical analysis on treatment effects with emphasis on the patients’ age at initiation of treatment, and no significant difference could be found. Suzuki and co-workers reported that the severity of the avascular necrosis had no relationship to the patient’s age at the beginning of treatment [18]. Kitoh and co-workers [19] reported that age had no statistical relationship with the failure of reduction and the incidence of avascular necrosis.

What then is the optimum age for treatment in the Pavlik harness? Currently, there is no clear answer to this question.

The mean age at initiation of Pavlik harness treatment at the 12 institutions participating in this study ranged from 3.5 to 4.8 months. In Japan, the majority of dysplastic hips are discovered in screening programs usually performed at 3 or 4 months of age. This may account for the relatively delayed initiation of treatment in this country. Also, many pediatric orthopedic surgeons have harbored the belief that very young patients may be more susceptible to the more severe types of avascular necrosis that lead to poor long-term outcomes. Whether this is true is also a question that requires clarification. There is a well-known study by Iwasaki of Nagasaki University School of Medicine [20]. Between 1966 and the first half of 1976, he treated 240 completely dislocated hips in 204 patients by using the Pavlik harness. The age of patients at the start of treatment ranged between 1 and 7 months. In Iwasaki’s investigation, reduction was achieved in 82.7 % of the children who were between 31 and 60 days at the beginning of the treatment. When the treatment began between 91 and 120 days of age, the reduction rate was an even better 91.1 %. Concerning avascular necrosis of the femoral head, it occurred in 13.6 % in the patients who started treatment by Pavlik harness between 31 and 60 days old, while only 6.3 % of patients whose initial treatment age was between 91 and 120 days after birth had avascular necrosis. From this evidence, it would seem that the optimum age for initiation of Pavlik harness treatment should be after reaching 91 days old, 3 months after birth in Japan. Overall the therapeutic results of this study are quite comparable with the results of the multicenter study of the European Pediatric Orthopaedic Society by Grill and co-workers [5]. However, differences in the patient population should be discussed. Grill and co-workers evaluated the reduction rate and avascular necrosis of the proximal femoral epiphysis according to the Tönnis grade. In the present study, we only addressed hips with complete dislocations, corresponding to Tönnis grade 3 and 4. In the multicenter study of Grill and co-workers, treatment with the Pavlik harness achieved reduction in 80 % of patients with dislocated hips. This rate was similar to our results, 80.2 % (1994) and 81.9 % (2008). Grill and co-workers in 1988 demonstrated that the rate of avascular necrosis with the Pavlik harness was 3.1 % in Tönnis grade 3 and 16.4 % in Tönnis grade 4. Our data on the incidence of avascular necrosis of the proximal femoral epiphysis for combined Tönnis grade 3 and 4 hips was 14.3 % in 1994 and 11.5 % in 2008. When comparing these European and Japanese results, racial difference could be another factor influencing outcome. It is reassuring to note that no major differences were observed.

Reduction rates and the incidence of avascular necrosis in the 2008 multicenter study were statistically similar to the results collected in 1994. However, the rate of acceptable outcome (Severin grade I + II/total) in 2008 was statistically higher than that in 1994.

We believe that the results of this study provide important insight into the present status of Pavlik harness therapy in our country. Application techniques for the harness were established by our forerunners more than 30 years ago, as shown by the unchanged reduction rates and unaltered incidence of avascular necrosis. Furthermore, statistically improved long-range outcomes could indicate that ongoing attention to treatment details are proving to be effective. One contributing factor to improved treatment outcomes over our study period could be the growing use of ultrasonography. Although physical examination is still the main tool in the diagnosis of infantile hip dislocation, many institutions now utilize ultrasonographic screening techniques, leading to improved diagnostic accuracy. In addition, ultrasonography is now used as a tool to monitor the progress of treatment with the Pavlik harness, usually through the anterior probing approach [21]. Attention to the details of Pavlik harness application has also led to better long-term results. Since the dangers of excessive abduction under the Pavlik harness have been reported [22], many institutions are using pillows or special supporting cushions to prevent this [20]. The Pavlik harness has now been the accepted initial treatment for infantile dislocated hips in Japan for many decades, with consistently satisfactory results in the majority of cases.

The rates of reduction in completely dislocated hips, the incidence of avascular necrosis complications and Severin X-ray changes in this report should be a bench mark for those undertaking treatment of the child’s hip with the Pavlik harness.

## Conclusion

First in 1994 and then in 2008, we sent questionnaires to multiple pediatric orthopedic institutions nationwide. We requested data concerning children treated with the Pavlik harness for developmental dysplasia of the hip. Our points of interest were the reduction rate and incidence of avascular necrosis of the femoral epiphysis, and in children followed to skeletal maturity at age 14 years, the X-ray outcomes. Reduction rates and the incidence of avascular necrosis in the 2008 multicenter study were statistically similar to the results collected in 1994. However, the rate of acceptable outcome (Severin grade I + II/total) in 2008 was statistically higher than that of 1994.
